# Bone Volumetric Density, Microarchitecture, and Estimated Bone Strength in Tumor-Induced Rickets/Osteomalacia *Versus* X-linked Hypophosphatemia in Chinese Adolescents

**DOI:** 10.3389/fendo.2022.883981

**Published:** 2022-06-13

**Authors:** Ruizhi Jiajue, Xiaolin Ni, Chenxi Jin, Wei Yu, Li Huo, Huanwen Wu, Yong Liu, Jin Jin, Wei Lv, Lian Zhou, Yu Xia, Yue Chi, Lijia Cui, Qianqian Pang, Xiang Li, Yan Jiang, Ou Wang, Mei Li, Xiaoping Xing, Xunwu Meng, Weibo Xia

**Affiliations:** ^1^ Department of Endocrinology, Key Laboratory of Endocrinology, National Commission of Health, State Key Laboratory of Complex Severe and Rare Diseases, Peking Union Medical College Hospital, Chinese Academy of Medical Science, Beijing, China; ^2^ Department of Radiology, Peking Union Medical College Hospital, Chinese Academy of Medical Sciences and Peking Union Medical College, Beijing, China; ^3^ Department of Nuclear Medicine, Peking Union Medical College Hospital, Chinese Academy of Medical Sciences and Peking Union Medical College, Beijing, China; ^4^ Department of Pathology, Peking Union Medical College Hospital, Chinese Academy of Medical Sciences and Peking Union Medical College, Beijing, China; ^5^ Department of Orthopedic Surgery, Peking Union Medical College Hospital, Chinese Academy of Medical Sciences and Peking Union Medical College, Beijing, China; ^6^ Department of Ear, Nose, and Throat, Peking Union Medical College Hospital, Chinese Academy of Medical Sciences and Peking Union Medical College, Beijing, China; ^7^ Department of Stomatology, Peking Union Medical College Hospital, Chinese Academy of Medical Sciences and Peking Union Medical College, Beijing, China; ^8^ Department of Ultrasound Diagnosis, Peking Union Medical College Hospital, Chinese Academy of Medical Sciences and Peking Union Medical College, Beijing, China

**Keywords:** tumor-induced rickets/osteomalacia, X-linked hypophosphatemia, high-resolution peripheral quantitative computed tomography, bone microarchitecture, estimated bone strength

## Abstract

Tumor-induced rickets/osteomalacia (TIR/O) severely impairs bone microarchitecture and bone strength. However, no study has described the microarchitectural quality of bone in adolescent patients with TIR/O. TIR/O affects bone quality more severely than the inherited causes of hypophosphatemia, the most common form of which is X-linked hypophosphatemia (XLH). Nevertheless, differences of the microarchitectural quality of the bone between TIR/O and XLH have never been clarified. Therefore, in this study, we used high-resolution peripheral quantitative computed tomography to assess bone microarchitecture in five Chinese adolescent TIR/O patients, and these were compared with 15 age- and gender-matched XLH patients as well as 15 age- and gender-matched healthy controls. Compared with the healthy controls, the TIR/O patients presented with significantly lower volumetric bone mineral densities (vBMDs), severely affected bone microarchitecture, and profoundly weaker bone strength. The distal tibia was more severely affected than the distal radius. Compared with the XLH patients, the TIR/O patients showed deteriorated bone quality notably at the distal tibia and in the cancellous compartment, reflected by 45.9% lower trabecular vBMD (*p* = 0.029), 40.2% lower trabecular fraction (*p* = 0.020), 40.6% weaker stiffness (*p* = 0.058), and 42.7% weaker failure load (*p* = 0.039) at the distal tibia. The correlation analysis showed that a higher level of serum FGF23 and a lower level of serum phosphate were associated with a poorer bone microarchitecture and a weaker estimated bone strength in the hypophosphatemic patients of our study. In conclusion, our study demonstrated significantly lower vBMDs, severely impaired bone microarchitecture, and profoundly weaker bone strength in Chinese adolescent patients with TIR/O, notably at the distal tibia, compared with the same parameters in age- and sex-matched healthy controls and XLH patients, which was possibly caused by excessive FGF23 production and secretion, chronically severe hypophosphatemia, and weak mechanical stimulus at the lower extremities. These findings further our understanding of the impact of different kinds of hypophosphatemic rickets/osteomalacia on bone quality.

## Introduction

Fibroblast growth factor 23 (FGF23)-related hypophosphatemic rickets/osteomalacia refers to a number of rare hereditary and acquired renal phosphate wasting disorders. The typical manifestations are chronic hypophosphatemia with inappropriately low or normal serum level of 1,25-dihydroxyvitamin D [1,25(OH)_2_D], defective bone mineralization with profound bone pain, and fragility fractures. X-linked hypophosphatemia (XLH) is the most common genetic cause of renal phosphate wasting disorder, which is caused by inactivating mutations in the phosphate-regulating gene with homologies to endopeptidases on the X chromosome (*PHEX*). Meanwhile, tumor-induced rickets/osteomalacia (TIR/O) is an acquired cause of FGF23-related hypophosphatemic disorder, which is caused by phosphaturic mesenchymal tumor (PMT) with excess production and secretion of FGF23 (1).

The severity of skeletal impairment can be dramatically different between XLH and TIR/O. Clinical manifestations, such as muscle weakness, bone pain, and fragility fractures, are more profound in TIR/O patients in comparison with the inherited causes of hypophosphatemia (2, 3). In addition, our previous study (4) showed that, compared with patients with XLH, patients with TIR/O had a significantly higher level of serum FGF23, a significantly lower level of serum phosphate, and a significantly lower level of areal bone mineral density (aBMD) as evaluated by dual-energy X-ray absorptiometry (DXA). Therefore, it seems that TIR/O patients suffer from a worse skeletal status than XLH patients. However, anatomical and anthropometric factors, such as hyperostosis, fracture, and vascular calcification, can interfere with DXA results (5). Therefore, aBMD may not reflect the actual skeletal status and must be interpreted with caution. Moreover, aBMD cannot discriminate between cortical and trabecular bone tissues and cannot estimate bone microarchitecture and strength.

High-resolution peripheral quantitative computed tomography (HR-pQCT) arises as a non-invasive imaging technique that is only slightly affected by the aforementioned interferences and allows the separate quantification of trabecular and cortical volumetric BMD (vBMD), bone geometry, and bone microarchitecture in the peripheral bone (6). In addition, using a software applied to the HR-pQCT images, finite element analysis (FEA) can be performed to estimate the bone strength (7). As a consequence, HR-pQCT analysis provides a better understanding of the impact of different metabolic bone diseases on bone quality.

Some studies have characterized bone microarchitectural parameters and bone strength in adult TIO patients (8, 9) and XLH patients (10–12) using HR-pQCT. However, no studies have evaluated the HR-pQCT parameters in adolescents with TIR/O. Moreover, differences of the microarchitectural quality of bones between TIR/O and XLH have never been clarified. Therefore, we conducted this study which is aimed to investigate the bone volumetric density, microarchitecture, and estimated bone strength in adolescents with TIR/O using HR-pQCT and FEA and to compare the HR-pQCT and FEA parameters between age- and gender-matched patients with TIR/O and XLH.

## Patients and Methods

### Patient Selection

We previously reported 10 Chinese adolescents (<20 years old) with TIR/O. Half of these TIR/O patients had their preoperative HR-pQCT measurements performed at Peking Union Medical College Hospital (PUMCH) between August 1, 2015 and August 31, 2020 and were included in this study (**Supplementary Table S1**). All these TIR/O patients reported a normal postoperative level of serum phosphate and a pathological confirmation of PMT (4). Two hundred sixty-one XLH patients with mutations in the *PHEX* gene were followed up for a long term at our institution since 2005. Weibo X et al. have previously reported the characteristics of these XLH patients (13). We selected age-matched (± 2 years) and gender-matched XLH patients who performed the HR-pQCT measurement at our institution during the same period in a 3:1 ratio of XLH to TIR/O.

The medical ethics committee of PUMCH has approved the study protocol. All subjects have signed the informed consent forms prior to their participation in this study.

### Clinical Investigation

All participants completed a questionnaire focused on the following clinical information: age at disease onset, age at surgery, and duration of medical treatment with calcitriol and phosphate salts. Age at disease onset was recalled by the patients or their relatives when they first noticed the abnormal manifestations. The height of each participant was measured using a wall-mounted stadiometer. Standard deviation score (SDS) for height was calculated using the standardized growth chart for Chinese children and adolescents (14). Body mass index (BMI) was calculated according to a standard formula (BMI = weight/height^2^).

### Laboratory Examinations

After overnight fasting, blood sample from each participant was collected between 7 and 9 am. The medications were discontinued at least 3 days before the blood samples were drawn from the patients being actively treated with phosphate salts and calcitriol. The blood samples of TIR/O patients were drawn 1–3 months before surgery. Auto-analyzer (Beckman Coulter, America) was adopted to measure the total calcium (Ca), serum phosphate (P), and alkaline phosphatase (ALP). Automated Roche electrochemiluminescence system (E170; Roche Diagnostics, Basel, Switzerland) was used to assess the serum C-terminal telopeptide of type I collagen (β-CTX), serum 25-hydroxyvitamin D [25(OH)D], and serum intact parathyroid hormone. Measurement of serum 1,25(OH)_2_D was conducted using an enzyme-linked immunoassay (DiaSorin, USA). A two-site enzyme-linked immunosorbent assay (ELISA) kit (KAINOS Laboratories, Inc., Tokyo, Japan) was used to assess the serum intact FGF23, the detectable concentration range of which is 3–800 pg/ml. Urine was analyzed by a urinary chemical analyzer (Clinitek 500, SIEMENS, USA) and a urine flow cytometer (Sysmex UF-1000i, Sysmex, Japan). The renal tubular reabsorption of phosphate (TRP) was calculated using the following formula: % TRP = 100 × [1 - (urine phosphate × serum creatinine)/(serum phosphate × urine creatinine)]. The tubular maximum reabsorption threshold of phosphate per glomerular filtration rate (TmP/GFR) was estimated using the Walton and bijvoet nomogram (15).

### Dual-Energy X-Ray Absorptiometry

aBMDs at the femoral neck, the great trochanter, and the lumbar spine (L1–L4) were assessed using the same DXA equipment (Lunar DPX; Lunar Corporation, Madison, WI, USA). The *Z*-scores were calculated based on the mean BMD and SDS for Chinese children and adolescents (16). The aBMDs of TIR/O patients were measured at 1–3 months before surgery.

### High-Resolution Peripheral Quantitative Computed Tomography

A HR-pQCT scanner (Xtreme CT; Scanco Medical AG, Switzerland) with an isotropic voxel size of 61 μm was adopted to assess the bone microarchitecture of each participant at the nondominant distal radius and corresponding distal tibia. Preoperative scans were performed for TIR/O patients at 1–3 months before surgery. We set the reference lines on the distal endplate of the scanned limbs. The scan built a three-dimensional representation by obtaining 168 CT slices at 9.0 mm proximal to the reference line for the distal radius and 22.0 mm for the distal tibia. The scanner automatically identified the periosteal bone surface, while the endosteal surface, the cortical compartment, and the trabecular compartment were identified using an Image Processing Language (v5.42, Scanco Medical) algorithm. The cross-sectional area of all compartments (Tt.Ar), cortical bone (Ct.Ar), and trabecular bone (Tb.Ar) as well as the vBMD of all compartments (Tt.vBMD), trabecular bone (Tb.vBMD), and cortical bone (Ct.vBMD) were directly measured. The trabecular bone parameters, such as fraction (Tb.BV/TV), number (Tb.N), thickness (Tb.Th), separation (Tb.Sp), and inhomogeneity (Tb.1/N.SD), as well as cortical bone parameters, including thickness (Ct.Th) and cortical porosity (Ct.Po), were calculated directly (17). Scanco Finite Element software (vision 1.13; Scanco Medical) was adopted to perform the micro-finite element analysis to calculate the estimated bone strength. The binary image was turned into a mesh of isotropic brick elements, and a uniaxial compression test was performed with a 1,000-N load and 1% apparent strain. A homogenous elastic modulus of 10 GPa and Poisson’s ratio of 0.3 were assigned to all elements. When 2% of the elements exceeded a local effective strain of 0.7%, failure load was assumed to occur (18).

Fifteen age- and gender-matched healthy control subjects were invited for HR-pQCT measurements from a cohort of 979 healthy people participating in a separate study at our center aimed at establishing HR-pQCT reference data in the Chinese population (19). None of these control subjects had any bone disease or were receiving any treatment that might affect bone metabolism.

### Statistical Analysis

The results were displayed as mean ± standard deviation for normally distributed data and median [interquartile range (IQR)] for non-parametric data. The distribution of quantitative data was verified using the Shapiro–Wilk test. Comparisons between groups were assessed using Student’s *t*-test or Welch’s *t*-test for normally distributed variables and Mann–Whitey *U*-test for non-parametric variables. Categorical data were shown as frequencies and percentages (%), and their comparisons between groups were analyzed using chi-square test and Fisher’s exact test. Bivariate correlations with adjustment for age and gender were analyzed using partial correlation analysis. The significance level was set at *p*-value <0.05 (two-tailed). All statistical analyses were performed using STATA statistical software (version 16.0, StataCorp LLC., College Station, TX, USA).

## Results

### Comparison of General Features, Biochemical Characteristics, and Bone Mineral Densities Between TIR/O and XLH in Chinese Adolescents

Five TIR/O patients (3 male and 2 female), with a mean age of 16.4 ± 2.1 years (range, 14–19 years), and 15 XLH patients (9 male and 6 female), with a mean age of 16.6 ± 3.3 years (range, 12–20 years), were enrolled. Four (80%) TIR/O patients presented with clinical and/or radiological signs of rickets, such as upped and flared metaphyses, widened and irregular physes of the long bones, rachitic rosary, hand and foot bracelets, and pectus carinatum. Six (40%) XLH patients, aged 19 to 20 years old with a disease duration of 210–223 months, presented with clinical and/or radiological signs of osteomalacia, such as pseudofractures, early osteoarthritis, and enthesopathies. Three XLH patients presenting with enthesopathy were overweight/obese (BMI >25 kg/m^2^). Comparisons of the general characteristics between these two hypophosphatemic disorders are detailed in **Table 1**. At the time of the study, all the TIR/O patients and about 70% of the XLH patients were being actively treated with calcitriol and phosphate salts. The XLH patients had a much younger onset age [15 (5) *versus* 120 (36) months, *p* = 0.001] and a longer course of medical treatment (180.1 ± 41.1 *versus* 58.6 ± 20.3 months, *p* < 0.001) than the TIR/O patients. In comparison with the XLH group, the TIR/O group showed a much higher serum level of FGF23 (521.08 ± 101.24 *versus* 105.61 ± 45.38 pg/ml, *p* = 0.003), a significantly lower serum level of phosphate (0.50 ± 0.08 *versus* 0.76 ± 0.22 mmol/L, *p* = 0.019), and a markedly evaluated serum level of ALP (991 ± 516 *versus* 351 ± 221 U/L, *p* = 0.048), whereas the serum β-CTX levels were not significantly different. Only four TIR/O patients (one without a measurement of FGF23) and three XLH patients had estimated TmP/GFR (**Supplementary Table S2**). Although we did not perform a statistical analysis due to the limited sample size, a higher FGF23 level seemed to correlate with a lower TmP/GFR value and a lower 1,25(OH)_2_D level, especially in the XLH patients. Furthermore, compared with XLH patients, the TIR/O patients seemed to have lower TmP/GFR values and lower 1,25(OH)_2_D_3_ levels, which might be due to their higher FGF23 levels. aBMDs at the great trochanter (*Z*-score, -1.6 ± 3.5 *versus* 1.4 ± 2.2, *p* = 0.075) and lumbar spine (*Z*-score, -3.3 ± 3.7 *versus* -0.3 ± 2.0, *p* = 0.067) were lower in the TIR/O group compared with the XLH group, and the values were approaching statistical significance.

**Table 1:** Comparison of general features, biochemical characteristics, and bone mineral densities between TIR/O and XLH.

Characteristics	TIR/O (*n* = 5)		XLH (*n* = 15)		P
	Results	Range	Results	Range	
Male/female	3:2	–	9:6	–	–
Age (years), mean ± SD	16.4 ± 2.1^a^	14–19	16.6 ± 3.3	12–20	0.900
Phosphate and calcitriol treatment, *n*/*N* (%)^b^	5/5 (100%)	–	15/15 (100%)	–	–
Current, *n*/*N* (%)	5/5 (100%)	–	11/15 (73.3%)	–	0.530
Previous, *n*/*N* (%)	0/5 (0%)	–	4/15 (26.7%)	–	0.530
Treatment duration (months), mean ± SD	58.6 ± 20.3	9–72	180.1 ± 41.1	36–192	**<0.001**
Age at disease onset (months), median (IQR)	120 (36)	96–168	15 (5)	6–60	**0.001**
Height SDS, mean ± SD	-1.8 ± 1.8	-3.7–0.7	-2.3 ± 1.4	-5.1–0.1	0.547
Weight (kg), mean ± SD	54.0 ± 13.7	40.0–76.0	49.8 ± 17.7	22.0–90.0	0.638
BMI (kg/m^2^), mean ± SD	21.99 ± 2.10	19.65–24.26	22.23 ± 4.90	13.01–30.42	0.918
Biochemical results^c^					
Serum FGF23 (pg/ml), mean ± SD	521.08 ± 104.24	374.72–618.19	105.61 ± 45.38	42.36–184.30	**0.003**
Serum phosphate (mmol/L), mean ± SD	0.50 ± 0.08	0.40–0.59	0.76 ± 0.22	0.46–1.20	**0.019**
Serum phosphate (×LLN, %), mean ± SD	53.9 ± 8.4	44.4–62.1	78.6 ± 22.0	51.1–126.3	**0.027**
Serum calcium (mmol/L), mean ± SD	2.32 ± 0.06	2.24–2.38	2.38 ± 0.11	2.21–2.60	0.325
Serum 25(OH)D (ng/ml), median (IQR)	19.3 (11.9)	5.7–26.5	15.6 (4.2)	7.2–39.5	0.853
Serum 1, 25(OH)_2_D3 (pg/ml), mean ± SD	17.10 ± 4.26	10.36–29.55	45.75 ± 6.87	15.37–86.65	**0.032**
Serum PTH (pg/ml), mean ± SD	85.7 ± 46.0	39.3–137.6	98.9 ± 56.9	23.0–205.0	0.649
Serum ALP (U/L), mean ± SD	991 ± 516	370–1,626	351 ± 211	94–727	**0.048**
Serum ALP (×ULN, %), mean ± SD	448.2 ± 257.4	215.1–824.6	158.5 ± 86.6	94.0–418.4	**0.001**
Serum β-CTX (ng/ml), mean ± SD	1.35 ± 0.70	0.84–2.39	1.77 ± 0.92	0.39–3.41	0.365
Bone mineral density^d^					
Femoral neck *Z*-score, median (IQR)	-0.7 ± 3.0	-4.1 - 2.0	0.7 ± 2.2	-1.2 - 5.3	0.314
Great trochanter *Z*-score, median (IQR)	-1.6 ± 3.5	-5.8 - 3.0	1.4 ± 2.2	-2.0 - 5.1	0.075
Lumbar spine *Z*-score, median (IQR)	-3.3 ± 3.7	-8.3 - -0.6	-0.3 ± 2.0	-2.5 - 3.2	0.067

TIR/O, tumor-induced rickets/osteomalacia; XLH, X-linked hypophosphatemia; IQR, interquartile range; SDS, standard deviation score; SD, standard deviation; FGF23, fibroblast growth factor 23; 25OHD, 25-hydroxyvitamin D; 1, 25(OH)_2_D3, 1,25-dihydroxyvitamin D3; PTH, parathyroid hormone; ALP, alkaline phosphatase; β-CTX, C-terminal telopeptide of type I collagen; ULN, upper limit of normal range; LLN, lower limit of normal range.

a Age at surgery for the TIR/O patients were shown.

b ”Current” refers to patients being actively treated, while “Previous” refers to patients who have discontinued the medication for at least 1 month before coming to our clinic. The mean duration of treatment was calculated based on data from patients previously and currently receiving calcitriol and phosphate supplementation.

c Calcitriol and phosphate supplementation were discontinued in a patient being actively treated 3 days before the biochemical data were collected. Blood samples of TIR/O patients were drawn 1–3 months before surgery. The reference range for FGF23 in our laboratory is 16.1–42.2 pg/ml ( ± 2 SD from the mean). Serum phosphate reference range according to age subgroups: 4–11 years old, 1.2–1.8 mmol/L; 12–15 years old, 0.95–1.75 mmol/L; over 15 years old, 0.9–1.5 mmol/L. Serum alkaline phosphatase reference range according to age and sex subgroups: 0–15 years old, 42–390 U/L; 16–18 years old, 52–171 U/L; ≥19 years old and male, 45–125 U/L; 19–49 years old and female, 35–100 U/L. The reference ranges of all the other biochemical parameters were obtained from the central laboratory of Peking Union Medical College Hospital: serum calcium, 2.13–2.7 mmol/L; 25OHD, 8–50 ng/ml; 1,25(OH)_2_D3, 19.6–54.3 pg/ml; PTH, 12–65 pg/ml; β-CTX, 0.26–0.512 ng/ml. The percent of the lower limit of the normal range of serum phosphate and the percent of the upper limit of the normal range of serum alkaline phosphatase were calculated and compared between TIR/O patients and XLH patients.

d The bone mineral densities of TIR/O patients were measured 1–3 months before surgery. Bold values indicate p value < 0.05.

### Comparison of HR-pQCT Measurements and Estimated Bone Strength

Nine male and six female patients with a mean age of 16.4 ± 2.6 years (range, 12–20 years), a mean height of 169.1 ± 7.2 cm (range, 155.0–178.0 cm), a mean weight of 63.8 ± 13.9 kg (range, 50.0–95.0 kg), and a mean BMI of 22.13 ± 3.39 kg/m^2^ (range, 19.47–29.98) were included as a healthy control group. The HR-pQCT measurements and data on estimated bone strength obtained from our hypophosphatemic patients and the healthy control subjects are detailed in **Tables 2**, **3**. The percentage differences of the HR-pQCT measurements and estimated bone strength between any two groups are demonstrated in **Supplementary Figure S1**.

**Table 2:** Comparison of HR-pQCT measurements and estimated bone strength at the distal radius.

	TIR/O (*n* = 5)	XLH (*n* = 15)	Control (*n* = 15)	*p*_TIR/O versus XLH_	*p*_TIR/O versus control_	*p*_XLH versus control_
Geometry						
Tt.Ar (mm^2^)	348.6 (107.7)	328.1 (159.9)	273.8 (92.4)	0.360	0.053	0.330
Tb.Ar (mm^2^)	313.4 (80.7)	279.6 (153.8)	212.4 (72.0)	0.275	**0.025**	0.093
Ct.Ar (mm^2^)	53.3 ± 15.2	46.5 ± 18.9	68.4 ± 14.4	0.479	0.061	**0.001**
Ct.Pm (mm)	88.2 ± 27.2	70.7 ± 11.7	70.4 ± 8.7	0.226	**0.033**	0.940
**Volumetric density**						
Tt.vBMD (mgHA/cm^3^)	222.9 ± 97.7	264.1 ± 73.9	335.9 ± 47.7	0.330	**0.003**	**0.004**
Tb.vBMD (mgHA/cm^3^)	156.9 ± 64.4	190.9 ± 62.6	155.4 ± 28.1	0.310	0.940	0.055
Ct.vBMD (mgHA/cm^3^)	558.8 ± 276.0	669.8 ± 161.9	918.1 ± 34.8	0.281	**<0.001**	**<0.001**
**Microstructure**						
Tb.N (1/mm)	1.124 ± 0.456	1.575 ± 0.466	1.358 ± 0.204	0.076	0.123	0.110
Tb.Th (mm)	0.258 ± 0.051	0.249 ± 0.024	0.234 ± 0.020	0.729	0.135	0.065
Tb.Sp (mm)	0.768 (0.246)	0.508 (0.270)	0.700 (0.101)	0.074	0.081	0.130
Tb.1/N.SD (μm)	0.401 (0.018)	0.224 (0.171)	0.263 (0.059)	**0.049**	**0.002**	0.576
Tb.BV/TV (%)	21.0 ± 11.2	28.1 ± 8.3	0.230 ± 0.041	0.147	0.537	**0.046**
Ct.Th (mm)	0.696 ± 0.351	0.739 ± 0.284	1.120 ± 0.170	0.783	**0.002**	**<0.001**
Ct.Po (%)	0.6 (0.8)	0.7 (1.2)	0.3 (0.1)	0.430	0.676	**0.046**
FEA						
Stiffness (kN/mm)	45.0 ± 40.6	61.4 ± 31.3	74.9 ± 16.4	0.363	**0.027**	0.155
Failure load (N)	2,487.5 ± 2,152.6	3,327.1 ± 1,669.3	4,124.5 ± 880.5	0.382	**0.023**	0.116

Data were presented as mean ± SD or median (interquartile range).

HR-pQCT, high-resolution peripheral quantitative computed tomography; TIR/O = tumor-induced rickets/osteomalacia; XLH, X-linked hypophosphatemia; Tt.Ar, total bone area; Tb.Ar, trabecular bone area; Ct.Ar, cortical bone area; Ct.Pm, cortical perimeter; HA, hydroxyapatite; Tt.vBMD, total bone mineral density; Tb.vBMD, trabecular bone mineral density; Ct.vBMD, cortical bone mineral density; Tb.N, trabecular number; Tb.Th, trabecular thickness; Tb.Sp, trabecular separation; Tb.1/N.SD, trabecular inhomogeneity; Tb.BV/TV, trabecular bone volume to total volume ratio, or trabecular fraction; Ct.Th, cortical thickness; Ct.Po, cortical porosity; FEA, finite element analysis. Bold values indicate p value < 0.05.

**Table 3:** Comparison of HR-pQCT measurements and estimated bone strength at the distal tibia.

	TIR/O (*n* = 5)	XLH (*n* = 15)	Control (*n* = 15)	*p*_TIR/O versus XLH_	*p*_TIR/O versus control_	*p*_XLH versus control_
Geometry						
Tt.Ar (mm^2^)	894.2 ± 380.1	717.4 ± 161.5	731.8 ± 169.9	0.364	0.195	0.814
Tb.Ar (mm^2^)	817.8 ± 380.5	593.8 ± 146.7	598.7 ± 153.6	0.262	0.075	0.930
Ct.Ar (mm^2^)	82.4 ± 25.6	129.1 ± 51.0	138.6 ± 30.8	0.068	**0.002**	0.539
Ct.Pm (mm)	119.5 ± 33.9	103.5 ± 12.0	104.9 ± 12.1	0.355	0.157	0.756
**Volumetric density**						
Tt.vBMD (mgHA/cm^3^)	157.6 ± 112.5	278.1 ± 72.4	328.3 ± 45.5	**0.012**	**<0.001**	**0.031**
Tb.vBMD (mgHA/cm^3^)	93.9 ± 76.6	167.2 ± 56.4	184.8 ± 36.2	**0.029**	**0.002**	0.317
Ct.vBMD (mgHA/cm^3^)	691.1 ± 251.7	796.3 ± 94.8	945.3 ± 39.5	0.409	**0.001**	**<0.001**
**Microstructure**						
Tb.N (1/mm)	0.802 ± 0.474	1.198 ± 0.384	1.350 ± 0.308	0.075	**0.007**	0.243
Tb.Th (mm)	0.252 (0.050)	0.273 (0.042)	0.267 (0.045)	0.238	0.861	0.119
Tb.Sp (mm)	1.802 ± 1.203	0.932 ± 0.366	0.738 ± 0.174	0.182	**0.003**	0.074
Tb.1/N.SD (μm)	0.598(2.254)	0.355 (0.316)	0.307 (0.133)	0.162	**0.033**	0.115
Tb.BV/TV (%)	15.4 ± 10.3	25.9 ± 7.1	0.279 ± 0.051	**0.020**	**0.002**	0.374
Ct.Th (mm)	0.636 (0.530)	1.455 (0.889)	1.529 (0.390)	**0.023**	**0.006**	0.384
Ct.Po (%)	1.0 ± 0.7	2.8 ± 1.9	1.3 ± 0.8	0.064	0.583	**0.010**
**FEA**						
Stiffness (kN/mm)	107.1 ± 68.2	175.8 ± 63.9	226.4 ± 56.7	0.058	**0.001**	**0.032**
Failure load (N)	5,508.8 ± 3,738.0	9.414.6 ± 3.234.8	12,257.9 ± 2,968.6	**0.039**	**0.001**	**0.020**

Data were presented as mean ± SD or median (interquartile range).

HR-pQCT, high-resolution peripheral quantitative computed tomography; TIR/O, tumor-induced rickets/osteomalacia; XLH, X-linked hypophosphatemia; Tt.Ar, total bone area; Tb.Ar, trabecular bone area; Ct.Ar, cortical bone area; Ct.Pm, cortical perimeter; HA, hydroxyapatite; Tt.vBMD, total bone mineral density; Tb.vBMD, trabecular bone mineral density; Ct.vBMD, cortical bone mineral density; Tb.N, trabecular number; Tb.Th, trabecular thickness; Tb.Sp, trabecular separation; Tb.1/N.SD, trabecular inhomogeneity; Tb.BV/TV, trabecular bone volume to total volume ratio, or trabecular fraction; Ct.Th, cortical thickness; Ct.Po, cortical porosity; FEA, finite element analysis. Bold values indicate p value < 0.05.

#### Comparing Each of the Hypophosphatemic Groups to the Healthy Control Group

Compared with the healthy controls, both the TIR/O patients and the XLH patients exhibited general impairments of geometry, vBMD, microarchitecture, and estimated bone strength, whereas the TIR/O patients seemed to have greater percentage differences of these HR-pQCT measurements and estimated bone strength in comparison with the XLH patients.

At the distal radius, patients with TIR/O showed 27.3% larger total bone area (*p* = 0.053), 47.6% larger trabecular bone area (*p* = 0.025), and 22.1% smaller cortical bone area (*p* = 0.061) with 25.3% longer cortical perimeter (*p* = 0.033) compared with the healthy controls (**Supplementary Figure S1B**). The total vBMD at the distal radius in patients with TIR/O was significantly lower than that in the healthy controls (-33.6%, *p* = 0.003), which was mainly caused by the significantly lower cortical vBMD (-39.1%, *p* < 0.001). Both the cortical and cancellous bone microarchitecture at the distal radius were impaired in TIR/O patients, reflected by 37.9% thinner cortical thickness (*p* = 0.002), 52.5% higher trabecular inhomogeneity (*p* = 0.002), and 9.7% marginally greater trabecular separation (*p* = 0.081) in comparison with healthy controls. The TIR/O patients had significantly weaker stiffness (-40.0%, *p* = 0.027) and failure load (-38.8%, *p* = 0.023) at the distal radius than the healthy controls.

At the distal tibia, although the total bone area was similar (22.2%, *p* = 0.195) and the difference of the trabecular bone area only reached marginal significance (36.6%, *p* = 0.075) between the TIR/O patients and the healthy controls, the TIR/O patients still had 40.5% significantly smaller cortical bone area than the healthy controls (*p* = 0.002). Compared with the healthy control subjects, the TIR/O patients had severe deterioration both in the trabecular compartment and in the cortical compartment, respectively reflected by 51.1% lower trabecular vBMD (*p* = 0.002), 40.6% lower trabecular number (*p* = 0.007), 44.8% lower trabecular fraction (*p* = 0.002), 144.2% higher trabecular separation (*p* = 0.003), and 94.8% higher trabecular inhomogeneity (*p* = 0.033) as well as by 26.9% lower cortical vBMD (*p* = 0.001) and 58.4% thinner cortical thickness (*p* = 0.006). Stiffness (-52.2%, *p* = 0.001) and failure load (-58.8%, *p* = 0.001) were also significantly weaker in the TIR/O patients compared with the healthy controls. As shown in **Supplementary Figure S1B**, the distal tibia was more affected than the distal radius in the TIR/O patients, reflected by the lower percentage differences of total vBMD (tibia *versus* radius: -52.0 *versus* -33.6%) and estimated bone strength (tibia *versus* radius: stiffness, -52.2 *versus* -40%; failure load, -55.8 *versus* -38.8%).

In comparison between the XLH group and the healthy control group, although we only found significantly weaker estimated bone strength at the distal tibia (stiffness -22.2%, *p* = 0.032; failure load -23.3%, *p* = 0.020) and not at the distal radius (stiffness -17.3%, *p* = 0.155; failure load -19.3%, *p* = 0.116), the percentage differences of estimated bone strength parameters at different skeletal sites were not markedly different, as can be seen in **Supplementary Figure S1C**. Moreover, the XLH patients had a significantly lower total vBMD both at the distal tibia (-15.3%, *p* = 0.031) and at the distal radius (-21.4%, *p* = 0.004) in comparison with the healthy subjects. Therefore, skeletal deterioration seemed comparable at different skeletal sites in the XLH patients. However, with respect to the healthy control group, the XLH group predominantly had significantly worse microarchitecture in the cortical compartment both at the distal radius (Ct.vBMD -27.1%, *p* < 0.001; Ct.Th -34.0%, *p* < 0.001; Ct. Po 133.3%, *p* = 0.046) and at the distal tibia (Ct.vBMD -15.8%, *p* < 0.001; Ct.Po 115.4%, *p* = 0.010), whereas it had variable results in the trabecular compartment (almost all *p* > 0.05, except for the trabecular fraction at the distal radius). Therefore, the cortical bone seemed to be more affected than the cancellous bone in the XLH patients.

#### Comparing the TIR/O Group to the XLH Group

At the distal radius, compared with the XLH group, the TIR/O group showed significant or marginally significant differences only in a few trabecular parameters (**Table 2** and **Supplementary Figure S1A**), such as lower trabecular number (-28.6%, *p* = 0.076), greater trabecular inhomogeneity (79.0%, *p* = 0.049), and larger trabecular separation (51.2%, *p* = 0.074).

However, at the distal tibia, both the cortical compartment and the cancellous compartment were more severely affected in the TIR/O patients compared with the XLH patients (**Table 3** and **Supplementary Figure S1A**), reflected by 43.3% lower total vBMD (*p* = 0.012), which was mainly caused by 45.9% lower trabecular vBMD (*p* = 0.029), 33.1% lower trabecular number (*p* = 0.075), 40.2% lower trabecular fraction (*p* = 0.020), and 56.3% thinner cortical thickness (*p* = 0.023), whereas the 65.0% lower cortical porosity (*p* = 0.064) might be due to 36.1% lower cortical area (*p* = 0.068). More importantly, stiffness (-40.6%, *p* = 0.058) and failure load (-42.7%, *p* = 0.039) at the distal tibia were significantly lower in the TIR/O patients in comparison with the XLH patients.

**Figure 1:**
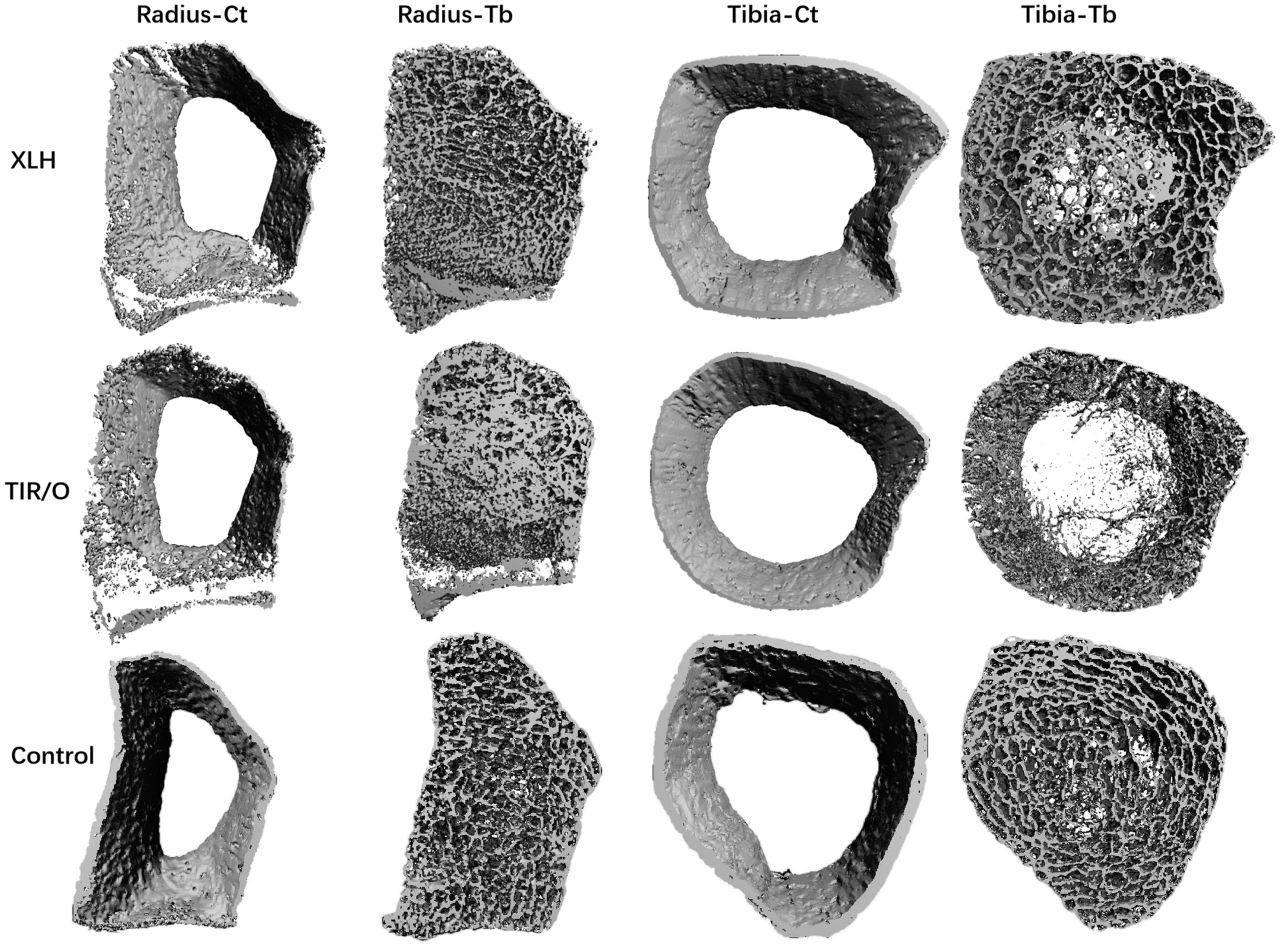
Representative bone images constructed by high-resolution peripheral quantitative computed tomography. The patient with TIR/O is an 18-year-old female subject with a height of 153 cm, the patient with XLH is an 18-year-old female subject with a height of 155 cm, and the healthy control is an 18-year-old female subject with a height of 160 cm. Note the clear differences in the trabecular network. Compared with the XLH patient and the healthy control, the trabecular bone microarchitecture is dramatically impaired in the TIR/O patient, especially at the distal tibia, with less trabecular number and larger trabecular separation. The cortical compartment is more affected at the distal radius in both the TIR/O patient and the XLH patient compared with the healthy control, with thinner cortical thickness and higher cortical porosity. TIR/O, tumor-induced rickets/osteomalacia; XLH, X-linked hypophosphatemia; Ct, cortical; Tb, trabecular.

Taken together, the TIR/O patients had a poorer skeletal status than the XLH patients, notably at the distal tibia and in the cancellous compartment. Representative reconstructed bone images from one TIR/O patient (18-year-old female patient) and an age- and gender-matched XLH patient (18-year-old female patient) are shown in **Figure 1**.

### Correlation Analysis of vBMD, Bone Microarchitecture, and Estimated Bone Strength With Biochemical Indices in Hypophosphatemic Patients

In order to test whether the abnormalities of biochemical parameters were associated with impaired bone microarchitecture in FGF23-mediated hypophosphatemic disorder, partial correlation analysis with adjustment for age and gender was performed to evaluate the correlations of HR-pQCT parameters (vBMD, bone microarchitecture, and estimated bone strength) with biochemical indices (serum FGF23, serum phosphate, and serum ALP) in the combined hypophosphatemic group (TIR/O + XLH, *n* = 20) (**Table 4**).

**Table 4:** Correlations of biochemical indices with HR-pQCT parameters in the combined hypophosphatemic group.

Variables	Serum FGF23		Serum phosphate		Serum ALP	
	*r*	*p*	*r*	*p*	*r*	*p*
Distal radius						
Total vBMD (mgHA/cm^3^)	-0.185	0.565	0.346	0.173	-0.386	0.126
Trabecular vBMD (mgHA/cm^3^)	-0.039	0.904	**0.482**	**0.050**	-0.410	0.102
Cortical vBMD (mgHA/cm^3^)	-0.375	0.230	0.039	0.883	-0.413	0.099
Trabecular number (1/mm)	-0.260	0.414	**0.591**	**0.013**	**-0.554**	**0.021**
Trabecular thickness (mm)	0.348	0.269	-0.017	0.948	-0.128	0.625
Trabecular separation (mm)	0.191	0.552	-0.445	0.074	**0.573**	**0.016**
Trabecular inhomogeneity (μm)	0.216	0.501	-0.396	0.115	**0.615**	**0.009**
Trabecular fraction (%)	-0.171	0.596	0.449	0.071	**-0.545**	**0.024**
Cortical thickness (mm)	-0.264	0.408	0.008	0.975	-0.168	0.519
Cortical porosity (%)	-0.216	0.501	-0.174	0.503	-0.145	0.579
Stiffness (kN/mm)	-0.373	0.259	0.364	0.166	-0.439	0.089
Failure load (N)	-0.370	0.263	0.352	0.182	-0.429	0.097
Distal tibia						
Total vBMD (mgHA/cm^3^)	**-0.599**	**0.040**	0.418	0.095	**-0.609**	**0.010**
Trabecular vBMD (mgHA/cm^3^)	-0.464	0.129	**0.645**	**0.005**	**-0.509**	**0.037**
Cortical vBMD (mgHA/cm^3^)	-0.368	0.240	0.073	0.780	**-0.608**	**0.010**
Trabecular number (1/mm)	-0.369	0.237	**0.645**	**0.005**	-0.423	0.091
Trabecular thickness (mm)	-0.455	0.138	-0.029	0.913	-0.303	0.237
Trabecular separation (mm)	0.432	0.161	**-0.540**	**0.025**	0.450	0.070
Trabecular inhomogeneity (μm)	0.470	0.123	-0.464	0.060	0.468	0.058
Trabecular fraction (%)	-0.490	0.106	**0.661**	**0.004**	**-0.554**	**0.021**
Cortical thickness (mm)	**-0.634**	**0.027**	0.208	0.424	**-0.598**	**0.011**
Cortical porosity (%)	-0.485	0.110	**0.532**	**0.028**	**-0.652**	**0.005**
Stiffness (kN/mm)	-0.575	0.064	**0.535**	**0.033**	**-0.572**	**0.021**
Failure load (N)	-0.555	0.077	**0.543**	**0.030**	**-0.607**	**0.013**

HR-pQCT, high-resolution peripheral quantitative computed tomography; FGF23, fibroblast growth factor 23; ALP, alkaline phosphatase; HA, hydroxyapatite; vBMD, volumetric bone mineral density. Bold values indicate p value < 0.05.

Serum FGF23 was negatively correlated with Tt.vBMD (*r* = -0.599, *p* = 0.040) and Ct.Th (*r* = -0.634, *p* = 0.027) at the distal tibia. Besides this, the negative correlation between serum FGF23 and estimated bone strength at the distal tibia also reached marginal significance (stiffness: *r* = -0.575, *p* = 0.065; failure load: *r* = -0.555, *p* = 0.077). Serum phosphate showed a significant correlation with many trabecular microarchitecture parameters both at the distal radius and the distal tibia, while it only showed a significantly positive correlation with estimated bone strength at the distal tibia (stiffness: *r* = 0.535, *p* = 0.033; failure load: *r* = 0.607, *p* = 0.013). At the distal radius, a higher serum ALP level was significantly correlated with a poorer trabecular microarchitecture, reflected by the positive correlations between serum ALP and trabecular separation (*r* = 0.573, *p* = 0.016) and inhomogeneity (*r* = 0.615, *p* = 0.009), and the negative correlations between serum ALP and trabecular number (*r* = -0.554, *p* = 0.0021) and fraction (*r* = -0.545, *p* = 0.024). At the distal tibia, a higher serum ALP level was significantly correlated with a lower vBMD in all the compartments and also correlated with a weaker estimated bone strength (**Table 4**).

We also conducted a partial correlation analysis in the XLH group (**Supplementary Table S3**). The correlations between biochemical indices and HR-pQCT parameters in the XLH group showed a similar trend with the results of the combined hypophosphatemic group. Although no significant correlation was identified between FGF23 and all the HR-pQCT parameters and between ALP and most of the HR-pQCT parameters, serum phosphate was still positively correlated with Tb.vBMD (*r* = 0.604, *p* = 0.038), Tb.N (*r* = 0.624, *p* = 0.030), and Tb.BV/TV (*r* = 0.618, *p* = 0.032), while it was negatively correlated with Tb.Sp (*r* = -0.648, *p* = 0.023) and Tb.1/N.SD (*r* = -0.669, *p* = 0.017). A partial correlation analysis was not conducted in the TIR/O group due to the small sample size (*n* = 5), the limited data of serum FGF23 (*n* = 4), and the variations of HR-pQCT parameters within the group. Nevertheless, our previous study in adult TIO patients reported similar correlations of FGF23 and ALP with the HR-pQCT parameters, while no significant correlation was found for serum phosphate (9).

## Discussion

This is the first study that describes the geometry, vBMD, bone microarchitecture, and estimated bone strength in adolescent patients with TIR/O. Compared with the healthy controls, our TIR/O patients had a higher total bone area, especially at the distal radius, which might arise as a geometric adaptation to preserve bone strength and improve bone resistance as previously reported in XLH (20). In addition, our TIR/O patients had a lower cortical bone area and a higher trabecular bone area both at the distal radius and tibia, referring to the cortex trabecularization in the peripheral skeleton of TIR/O patients (9).

Consistent with previous studies conducted in adult patients with TIO (8, 9, 21), our adolescent patients with TIR/O also presented with significantly lower vBMDs and severely affected bone microarchitecture in comparison with the healthy controls. However, we reported a larger percentage difference of most of the HR-pQCT parameters and estimated bone strength than the previous studies (8, 9). This discrepancy may be due to the early onset age [median (IQR), 120 (36) months] and the relatively long disease duration [median (IQR), 60 (31) months] of our TIR/O patients. Unlike vBMDs, differences of aBMDs between groups did not reach statistical significance, which might be caused by the huge variations within groups, further indicating that aBMD is not an ideal parameter to evaluate osteomalacia.

Compared with the healthy controls, our Chinese adolescents with TIR/O showed significantly impaired failure load and bone stiffness at both the distal radius and the distal tibia. Estimated bone strength has been proven to be associated with fragility fractures (22). Two of our five TIR/O patients indeed experienced fragility fractures at the femoral neck and lower extremities (4).

In agreement with the findings of Zanchetta MB et al. **(** 8 **)** and Ni X *et al.* (9), the HR-pQCT parameters and estimated bone strength were less affected at the distal radius than at the distal tibia in our TIR/O patients, which might be attributable to the distinct mechanical load at each skeletal site. The TIO patients commonly present with severe mobility impairment and more fragility fractures in the lower extremities, with most patients requiring wheelchairs or crutches (2). Consequently, the mechanical load in the lower extremities (including the distal tibia) should be weaker than the mechanical load in the upper extremities (including the distal radius). Ni X et al. **(** 9 **)** have further proven that indeed more differential HR-pQCT parameters at the distal tibia, not at the distal radius, were found between patients with different mobility impairments and between patients with and without a fracture history.

We also conducted the first study directly comparing the HR-pQCT parameters and estimated bone strength between age- and gender-matched patients with TIR/O and XLH. Our adolescent patients with TIR/O manifested with a poorer skeletal status than the XLH patients, notably at the distal tibia and in the cancellous compartment. These findings provide a reasonable explanation for the more profound skeletal pain and more severe fractures in TIO patients compared with the patients with hereditary hypophosphatemia (2). We also reported that a significantly higher serum FGF23 level and a significantly lower serum phosphate level were associated with TIR/O patients compared with XLH patients, which probably cause the huge differences of skeletal status between these two hypophosphatemic disorders. FGF23 is an endocrine hormone produced primarily in the bone and acts as a systemic phosphaturic factor that inhibits renal phosphate reabsorption by downregulation of sodium-phosphate cotransporter expression in the proximal tubule of the kidney (23). In addition, FGF23 also impairs the production of 1,25(OH)_2_D through inhibition of 1α-hydroxlase and stimulation of 24-hydroxylase, which further exacerbates hypophosphatemia (24). Therefore, high FGF23 levels are associated with worse hypophosphatemia, which then leads to severe defects of bone mineralization in FGF23-mediated hypophosphatemic rickets/osteomalacia. The correlation analysis did show that a higher serum FGF23 level was associated with a lower total vBMD, a thinner cortical thickness, and a weaker estimated bone strength at the distal tibia and that a lower serum phosphate level was associated with a lower trabecular vBMD, an impaired trabecular microarchitecture, and a weaker estimated bone strength at the distal tibia. In line with our findings, a correlation between high FGF23 and defective bone microarchitecture has been shown in either adult TIO patients (9) or osteoporotic patients (25), and severe hypophosphatemia has also been shown to cause a compromised bone microarchitecture and bone strength in adult XLH patients (12). In addition to its phosphate-regulating role, FGF23 may have a direct effect on bone mineralization and, consequently, on bone microarchitecture. Rupp T et al.^25^) showed that a high FGF23 level was associated with an impaired trabecular bone microarchitecture in osteoporotic patients, independently of serum phosphate and 25OHD. Wang H et al. (26) have proven that FGF23 overexpression not only impaired osteoblast differentiaon but also suppressed matrix mineralization, independently of its systemic effect of phosphate homeostasis. Therefore, the higher serum FGF23 level, the more severe hypophosphatemia is, and the weaker mechanical load at the lower extremities in the TIR/O patients possibly caused their extremely poorer skeletal status than the XLH patients.

The main inevitable limitations of our study are its retrospective design and the small sample size, but the rarity of TIR/O in adolescents must be considered. Usually for bone mineral density and bone microarchitecture measurements, the analyses should be performed separately for male and female individuals. However, due to the small sample size, separate analyses seemed unpractical. Moreover, the HR-pQCT scan position is determined by the fixed offset method, which does not take body size into account. In populations with a smaller short stature, such as the hypophosphatemic patients, the use of fixed offset method results in relatively proximal images (27, 28). Therefore, comparisons of HR-pQCT parameters between patients with different heights should be interpreted with caution. In addition, all the patients were evaluated at a reference center; therefore, they might present with more severe manifestations (referral bias). Due to these limitations, a larger multicenter study using relative offset method to determine the HR-pQCT scan position needs to be conducted in the future to validate our results.

In conclusion, our study demonstrated significantly lower vBMDs, severely impaired bone microarchitecture, and profoundly weaker bone strength in Chinese adolescent patients with TIR/O, notably at the distal tibia, compared with the same parameters in age- and gender-matched healthy controls and XLH patients, which was possibly caused by excessive FGF23 production and secretion, chronic severe hypophosphatemia, and the lack of a mechanical stimulus at the lower extremities. These findings further our understanding of the impact of different kinds of hypophosphatemic rickets/osteomalacia on bone quality.

## Data Availability Statement

The raw data supporting the conclusions of this article will be made available by the authors without undue reservation.

## Ethics Statement

The studies involving human participants were reviewed and approved by the Department of Scientific Research, the ethics committee in Peking Union Medical College Hospital. Written informed consent to participate in this study was provided by the participants’ legal guardian/next of kin.

## Author Contributions

RJ and WX designed the study protocol. RJ drafted the manuscript. CJ, XN, and RJ collected the data. RJ conducted the statistical analysis and interpreted the results. WX, YJ, OW, ML, and XX revised the manuscript. RJ and WX are responsible for the integrity of the data analysis. All authors contributed to the article and approved the submitted version.

## Conflict of Interest

The authors declare that the research was conducted in the absence of any commercial or financial relationships that could be construed as a potential conflict of interest.

## Publisher’s Note

All claims expressed in this article are solely those of the authors and do not necessarily represent those of their affiliated organizations, or those of the publisher, the editors and the reviewers. Any product that may be evaluated in this article, or claim that may be made by its manufacturer, is not guaranteed or endorsed by the publisher.

## Supplementary Material

Supplementary Figure 1 Percentage differences of the mean or median high-resolution peripheral quantitative computed tomography parameters in Chinese adolescents. **(A)** TIR/O versus XLH using the XLH group as the reference group. (**B**) TIR/O versus healthy control using the healthy control group as the reference group. **(C)** XLH versus healthy control using the healthy control group as the reference group. TIR/O, tumor-induced rickets/osteomalacia; XLH, X-linked hypophosphatemia.
