# Supplementary figures and images for: Bone Volumetric Density, Microarchitecture, and Estimated Bone Strength in Tumor-Induced Rickets/Osteomalacia Versus X-linked Hypophosphatemia in Chinese Adolescents
Source: Front Endocrinol (Lausanne). 2022 Jun 13;13:883981. doi: 10.3389/fendo.2022.883981 (PMC9234144; doi:10.3389/fendo.2022.883981)

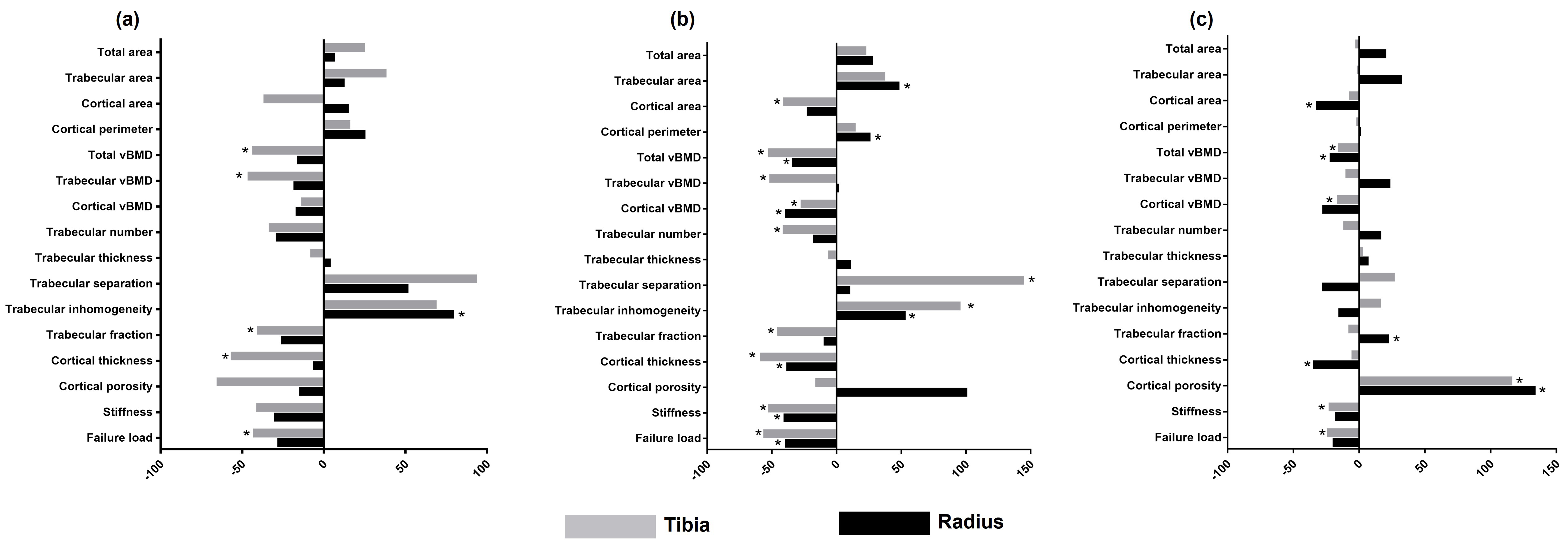

Supplement: Supplementary Figure 1 — Percentage differences of the mean or median high-resolution peripheral quantitative computed tomography parameters in Chinese adolescents. (A) TIR/O versus XLH using the XLH group as the reference group. (B) TIR/O versus healthy control using the healthy control group as the reference group. (C) XLH versus healthy control using the healthy control group as the reference group. TIR/O, tumor-induced rickets/osteomalacia; XLH, X-linked hypophosphatemia. [file Image_1.jpeg]
