# Supplementary material for: Bone Volumetric Density, Microarchitecture, and Estimated Bone Strength in Tumor-Induced Rickets/Osteomalacia Versus X-linked Hypophosphatemia in Chinese Adolescents
Source: Front Endocrinol (Lausanne). 2022 Jun 13;13:883981. doi: 10.3389/fendo.2022.883981 (PMC9234144; doi:10.3389/fendo.2022.883981)
Supplement: Supplementary file 2 [file Table_1.docx]

**Supplementary Table 1. Characteristics of 5 Chinese children and adolescents with tumor induced rickets/osteomalacia**

| **Characteristics** |  | **Cases with tumor induced rickets/osteomalacia** | | | | |
| --- | --- | --- | --- | --- | --- | --- |
| Gender/age at surgery (years) |  | F/15 | F/18 | M/14 | M/16 | M/19 |
| Age at disease onset (years) |  | 10 | 14 | 10 | 8 | 13 |
| Duration before surgery (months) |  | 60 | 41 | 36 | 84 | 72 |
| Height (cm) (SDS) |  | 141 (-3.4) | 153 (-1.4) | 158 (-1.1) | 149 (-3.7) | 177 (+0.7) |
| Weight (kg) |  | 40 | 46 | 55 | 23.87 | 76 |
| Body mass index (kg/m^2^) |  | 20.12 | 19.65 | 22.03 | 8 | 24.20 |
| Phosphate and calcitriol treatment |  | Current | Current | Current | Current | Current |
| Treatment duration (months) |  | 9 | 28 | 30 | 72 | 70 |
| ***Biochemical results before surgery*** |  |  |  |  |  |  |
| Serum phosphate (mmol/L) |  | 0.43 | 0.40 | 0.59 | 0.53 | 0.53 |
| TmP/GFR (mmol/L) |  | 0.48 | 0.50 | NA | 0.44 | 0.31 |
| Serum FGF23 (pg/mL) |  | NA | 529.41 | 562.01 | 374.72 | 618.19 |
| Serum calcium (mmol/L) |  | 2.34 | 2.24 | 2.37 | 2.28 | 2.38 |
| Serum 25(OH)D (ng/mL) |  | 26.5 | 22 | 10.1 | 5.7 | 19.3 |
| Serum 1, 25(OH)_2_D3 (pg/mL) |  | 13.62 | 14.88 | NA | 10.36 | 29.55 |
| Serum PTH (pg/mL) |  | 127 | 82.6 | 137.6 | 39.3 | 41.9 |
| Serum ALP (U/L) |  | 839 | 370 | 1626 | 1410 | 710 |
| Serum β-CTX (ng/mL) |  | 0.9 | 0.849 | 0.844 | 2.39 | 1.74 |
| ***Bone mineral density before surgery*** |  |  |  |  |  |  |
| Femoral neck BMD (g/cm^2^) |  | 0.408 | 0.468 | 0.954 | 0.766 | 1.242 |
| Femoral neck Z-score |  | -4.1 | -3.8 | 0.5 | -1.0 | 2.0 |
| Great trochanter BMD (g/cm^2^) |  | 0.291 | 0.157 | 0.650 | 0.413 | 1.076 |
| Great trochanter Z-score |  | -4.1 | -5.8 | 0 | -2.1 | 3.0 |
| Lumbar spine BMD (g/cm^2^) |  | 0.568 | 0.541 | 1.018 | 0.905 | 1.257 |
| Lumbar spine Z-score |  | -6.4 | -8.3 | -0.6 | -2.7 | -0.7 |
| ***HR-pQCT parameters at the distal radius before surgery*** |  |  |  |  |  |  |
| Total vBMD (mgHA/cm^3^) |  | 314.7 | 195.7 | 220.5 | 75.5 | 308.1 |
| Trabecular vBMD (mgHA/cm^3^) |  | 124.0 | 165.7 | 188.3 | 68.3 | 238.2 |
| Cortical vBMD (mgHA/cm^3^) |  | 916.2 | 461.7 | 498.7 | 190.5 | 727.1 |
| Trabecular number (1/mm) |  | 0.995 | 1.283 | 1.470 | 0.389 | 0.995 |
| Trabecular thickness (mm) |  | 0.231 | 0.260 | 0.261 | 0.200 | 0.231 |
| Trabecular separation (mm) |  | 0.958 | 0.768 | 0.712 | 2.618 | 0.640 |
| Trabecular inhomogeneity (μm) |  | 0.401 | 0.383 | 0.401 | 1.884 | 0.274 |
| Trabecular fraction (%) |  | 19.4 | 23.7 | 22.9 | 3.8 | 35.0 |
| Cortical thickness (mm) |  | 1.213 | 0.441 | 0.537 | 0.391 | 0.898 |
| Cortical porosity (%) |  | 0.1 | 0.6 | 0.9 | 0.1 | 1.1 |
| Stiffness (kN/mm) |  | 78.1 | 26.5 | 22.9 | 0.6 | 96.9 |
| Failure load (N) |  | 4333.4 | 1523.4 | 1369.4 | 64.1 | 5147.3 |
| ***HR-pQCT parameters at the distal tibia before surgery*** |  |  |  |  |  |  |
| Total vBMD (mgHA/cm^3^) |  | 286.8 | 61.3 | 103.8 | 64.3 | 272.0 |
| Trabecular vBMD (mgHA/cm^3^) |  | 128.6 | 8.8 | 74.5 | 50.9 | 206.9 |
| Cortical vBMD (mgHA/cm^3^) |  | 943.0 | 820.4 | 616.3 | 292.2 | 783.6 |
| Trabecular number (1/mm) |  | 0.963 | 0.328 | 0.926 | 0.342 | 1.450 |
| Trabecular thickness (mm) |  | 0.312 | 0.186 | 0.238 | 0.252 | 0.288 |
| Trabecular separation (mm) |  | 1.028 | 3.103 | 1.127 | 3.108 | 0.645 |
| Trabecular inhomogeneity (μm) |  | 0.420 | 2.674 | 0.598 | 2.702 | 0.243 |
| Trabecular fraction (%) |  | 20.3 | 4.2 | 12.3 | 9.8 | 30.6 |
| Cortical thickness (mm) |  | 1.351 | 0.636 | 0.478 | 0.398 | 1.008 |
| Cortical porosity (%) |  | 1.0 | 0.8 | 0.9 | 0.2 | 2.2 |
| Stiffness (kN/mm) |  | 148.5 | 53.6 | 53.3 | 73.2 | 207.0 |
| Failure load (N) |  | 7951.9 | 2943.7 | 3192.2 | 2544.7 | 10911.6 |

SDS = standard deviation score; NA = not applicable; TMP/GFR = tubular maximum reabsorption of phosphate/glomerular filtration rate; FGF23 = fibroblast growth factor 23; 25OHD = 25-hydroxyvitamin D; 1, 25(OH)_2_D3 = 1,25-dihydroxyvitamin D3; PTH = parathyroid hormone; ALP = alkaline phosphatase; β-CTX = C-terminal telopeptide of type I collagen; BMD = bone mineral density.

**Supplementary Table 2. TmP/GFR in some TIR/O patients and XLH patients**

|  | TIR/O | | | | |  | XLH | | | |
| --- | --- | --- | --- | --- | --- | --- | --- | --- | --- | --- |
|  | F/15 | F/18 | M/15 | M/19 | Mean |  | F/12 | F/15 | M/16 | Mean |
| TmP/GFR (mmol/L) | 0.48 | 0.50 | 0.44 | 0.31 | 0.43 |  | 0.41 | 0.68 | 0.45 | 0.51 |
| Serum phosphate (mmol/L) | 0.43 | 0.40 | 0.53 | 0.53 | 0.47 |  | 0.45 | 0.84 | 0.53 | 0.61 |
| Serum 1,25(OH)_2_D_3_ (pg/ml) | 13.62 | 14.88 | 10.36 | 29.55 | 17.10 |  | 46.38 | 70.59 | 52.26 | 56.41 |
| Serum FGF23 (pg/ml) | NA | 529.41 | 374.72 | 618.19 | 507.44 |  | 181.46 | 47.30 | 100.75 | 109.84 |

TIR/O = tumor-induced rickets/osteomalacia; XLH = X-linked hypophosphatemia; TmP/GFR = renal tubular maximum transport of phosphate (TmP) to glomerular filtration rate (GFR) ratio; 1, 25(OH)_2_D3 = 1,25-dihydroxyvitamin D3; FGF23 = fibroblast growth factor 23.

**Supplementary Table 3. Correlations of biochemical indices with HR-pQCT parameters in the XLH patients**

| **Variables** | **Serum FGF23** | | **Serum phosphate** | | **Serum ALP** | |
| --- | --- | --- | --- | --- | --- | --- |
|  | ***r*** | ***p*** | ***r*** | ***p*** | ***r*** | ***p*** |
| ***Distal radius*** |  |  |  |  |  |  |
| Total vBMD (mgHA/cm^3^) | 0.175 | 0.679 | 0.393 | 0.206 | -0.288 | 0.365 |
| Trabecular vBMD (mgHA/cm^3^) | -0.131 | 0.758 | 0.479 | 0.115 | -0.157 | 0.626 |
| Cortical vBMD (mgHA/cm^3^) | 0.520 | 0.186 | -0.216 | 0.499 | -0.637 | **0.026** |
| Trabecular number (1/mm) | -0.087 | 0.837 | 0.534 | 0.074 | -0.232 | 0.468 |
| Trabecular thickness (mm) | -0.306 | 0.462 | -0.024 | 0.941 | 0.058 | 0.857 |
| Trabecular separation (mm) | 0.030 | 0.943 | -0.526 | 0.079 | 0.170 | 0.598 |
| Trabecular inhomogeneity (μm) | -0.039 | 0.926 | -0.541 | 0.070 | 0.194 | 0.546 |
| Trabecular fraction (%) | -0.134 | 0.751 | 0.401 | 0.197 | -0.182 | 0.571 |
| Cortical thickness (mm) | 0.413 | 0.310 | 0.079 | 0.808 | -0.250 | 0.434 |
| Cortical porosity (%) | 0.013 | 0.976 | -0.413 | 0.183 | 0.589 | **0.044** |
| Stiffness (kN/mm) | -0.157 | 0.737 | 0.470 | 0.144 | -0.039 | 0.908 |
| Failure load (N) | -0.134 | 0.774 | 0.472 | 0.143 | -0.045 | 0.896 |
| ***Distal tibia*** |  |  |  |  |  |  |
| Total vBMD (mgHA/cm^3^) | 0.383 | 0.349 | 0.236 | 0.459 | -0.077 | 0.811 |
| Trabecular vBMD (mgHA/cm^3^) | -0.107 | 0.801 | 0.604 | **0.038** | -0.055 | 0.866 |
| Cortical vBMD (mgHA/cm^3^) | 0.666 | 0.071 | -0.389 | 0.216 | -0.524 | 0.080 |
| Trabecular number (1/mm) | -0.245 | 0.560 | 0.624 | **0.030** | -0.033 | 0.919 |
| Trabecular thickness (mm) | 0.354 | 0.390 | -0.311 | 0.325 | 0.105 | 0.746 |
| Trabecular separation (mm) | 0.160 | 0.704 | -0.648 | **0.023** | 0.005 | 0.988 |
| Trabecular inhomogeneity (μm) | 0.108 | 0.798 | -0.669 | **0.017** | -0.043 | 0.895 |
| Trabecular fraction (%) | -0.124 | 0.769 | 0.618 | **0.032** | -0.066 | 0.838 |
| Cortical thickness (mm) | 0.565 | 0.145 | -0.107 | 0.742 | -0.043 | 0.894 |
| Cortical porosity (%) | -0.089 | 0.834 | 0.367 | 0.241 | -0.133 | 0.680 |
| Stiffness (kN/mm) | 0.140 | 0.766 | 0.441 | 0.174 | 0.424 | 0.194 |
| Failure load (N) | 0.150 | 0.749 | 0.459 | 0.155 | 0.405 | 0.216 |

HR-pQCT = high-resolution peripheral quantitative computed tomography; XLH = X-linked hypophosphatemia; FGF23 = fibroblast growth factor 23; ALP = alkaline phosphatase; HA = hydroxyapatite; vBMD = volumetric bone mineral density.
